# Supplementary material for: Genome-wide perturbations of Alu expression and Alu-associated post-transcriptional regulations distinguish oligodendroglioma from other gliomas
Source: Commun Biol. 2022 Jan 18;5:62. doi: 10.1038/s42003-022-03011-w (PMC8766575; doi:10.1038/s42003-022-03011-w)
Supplement: Supplementary file 2 — Supplementary Information [file 42003_2022_3011_MOESM2_ESM.pdf]

Genome-wide perturbations of Alu expression and Alu-associated post-transcriptional regulations distinguish oligodendroglioma from other gliomas

Taeyoung Hwang, Sojin Kim, Tamrin Chowdhury, Hyeon Jong Yu, Kyung-Min Kim, Ho Kang, Jae Kyung Won, Sung-Hye Park, Joo Heon Shin, Chul-Kee Park

Supplementary Figure 1. The proportions of A-to-I editing sites that were shared by patients. Proportion (y-axis) is the number of A-to-I editing sites observed by the specified number of patients (x-axis) divided by the total number of A-to-I editing sites (572,385). The numbers of A-to-I editing sites found in 39, 40 and 41 patients were labelled for details.

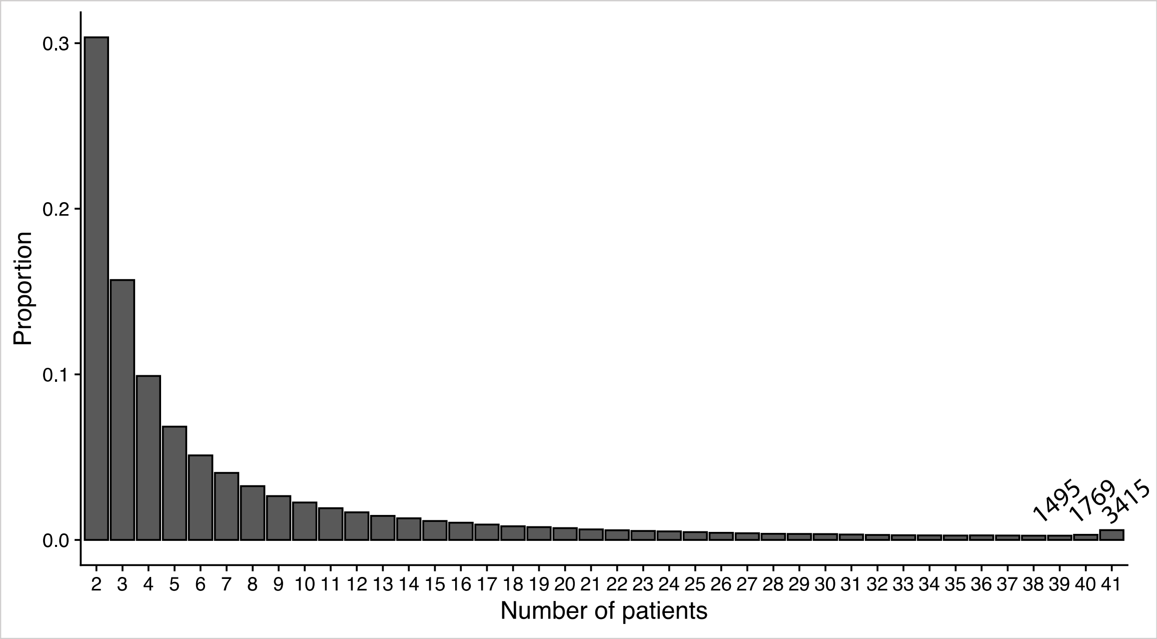

Supplementary Figure 2. Clustering of samples with 3415 common A-to-I editing sites. A heatmap was generated with the 3415 sites shared by all the patients (see Supplementary Figure 1). The distances between samples were measured by Pearson correlation coefficients of A-to-I editing levels. Hierarchical clustering was performed with Ward's method. A row is a A-to-I editing site and a column is a sample. Gradient colors from blue to red indicate A-to-I editing levels (unit: a rate of read counts from 0 to 1), defined as the ratio of inosine-supporting read counts relative to the adenosine or inosine-supporting read counts. The label of tumor or normal tissue is represented by green or black in an indication bar above the heatmap.

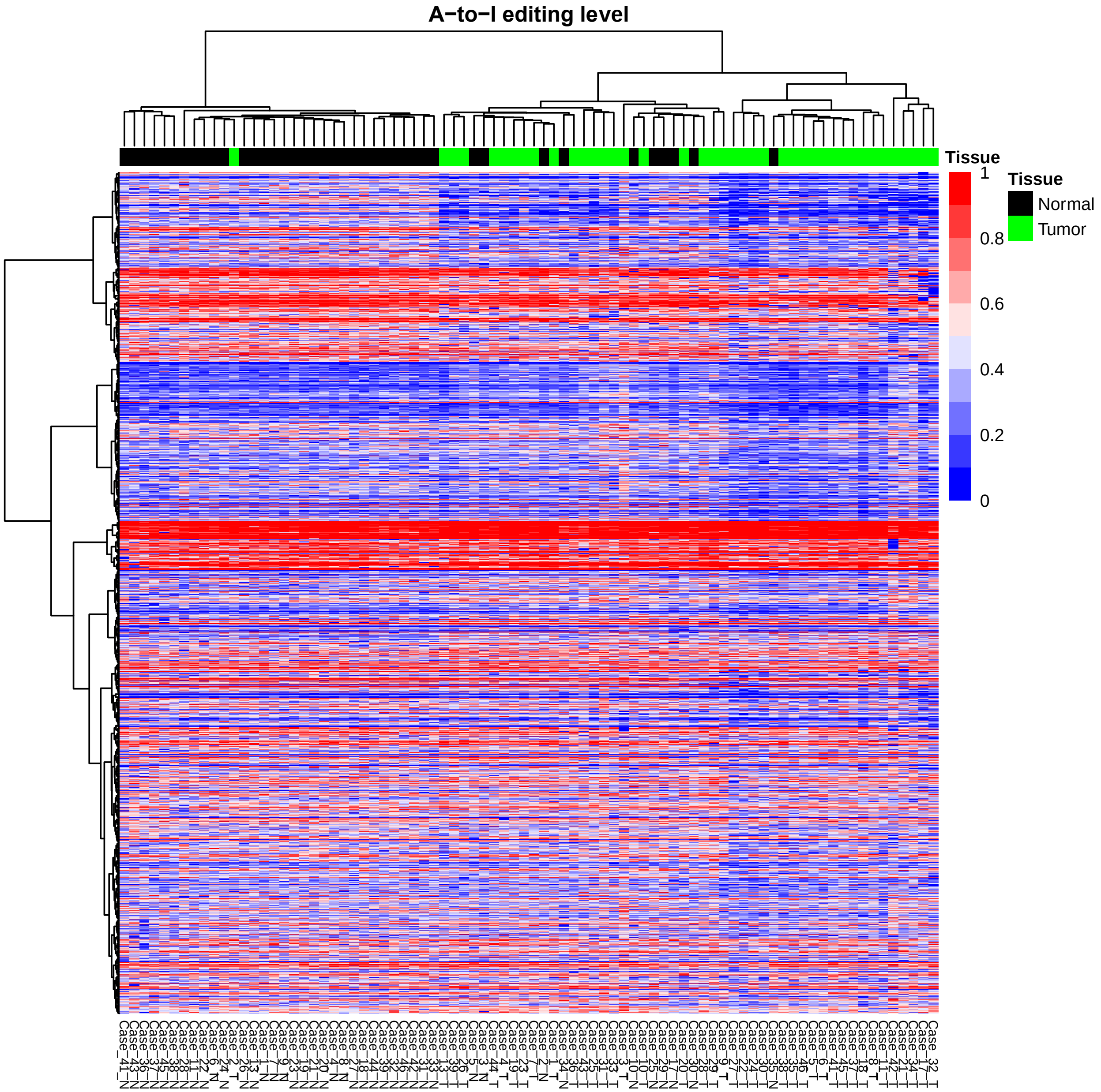

Supplementary Figure 3. Clustering analysis of samples. t-SNE (t-distributed stochastic neighbor embedding) analysis was performed with gene expression levels (TPM) to check clustering of the samples in the current dataset. Glioblastoma (GBM) and grade 2 or 3 oligodendrogliomas (O2 or O3) are located at distinct regions of the plot in general.

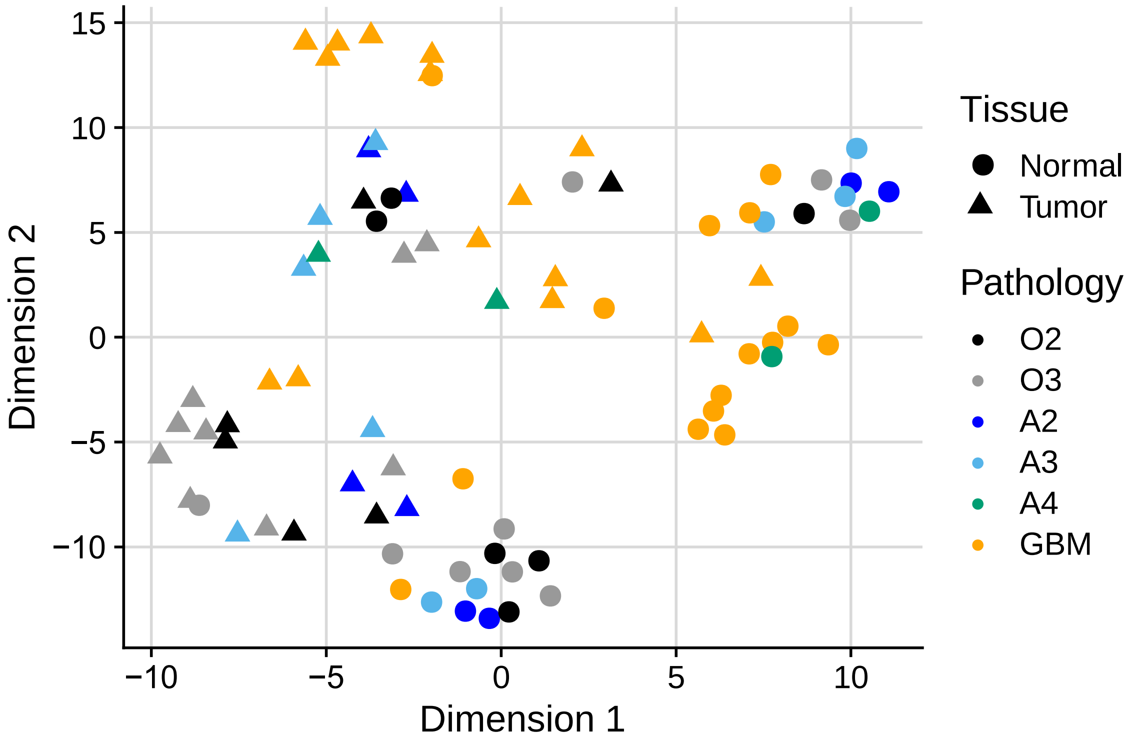

Supplementary Figure 4. Overlaps of differentially-edited A-to-I editing sites between two pathologies. After differentially-edited A-to-I editing sites between tumor and the matched normal tissues were identified per pathology, the proportions of the overlapped sites relative to the smaller numbers in the two comparing pathologies were calculated. Color gradation is proportional to the size of overlaps.

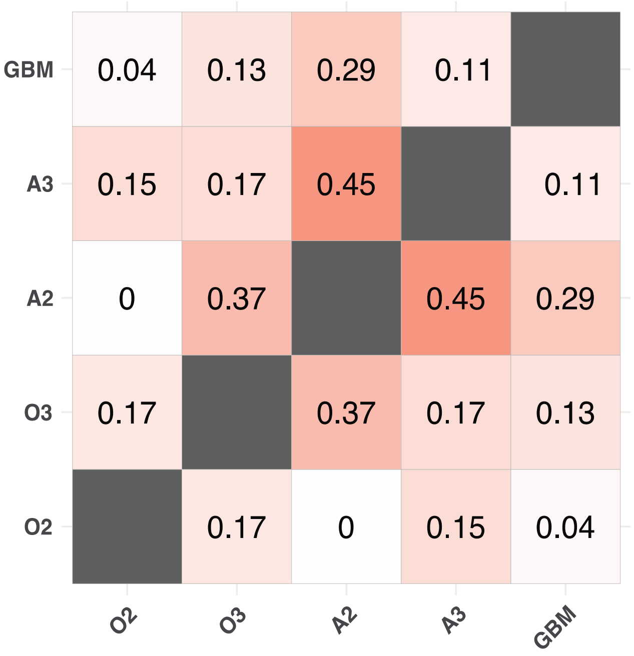

Supplementary Figure 5: Alu Editing Index (AEI) comparison between tumor and normal tissues per pathology. The p-values calculated by Wilcoxon rank sum test (two-sided) were shown below the names of pathologies. Each dot is a tissue (N=12, 18, 8, 10, 30 tissues for O2, O3, A2, A3, GBM, respectively). In a boxplot, the whiskers extend from the bottom and top of the box (the first and third quartiles) to the largest and the smallest value no further than 1.5 * inter-quartile range.

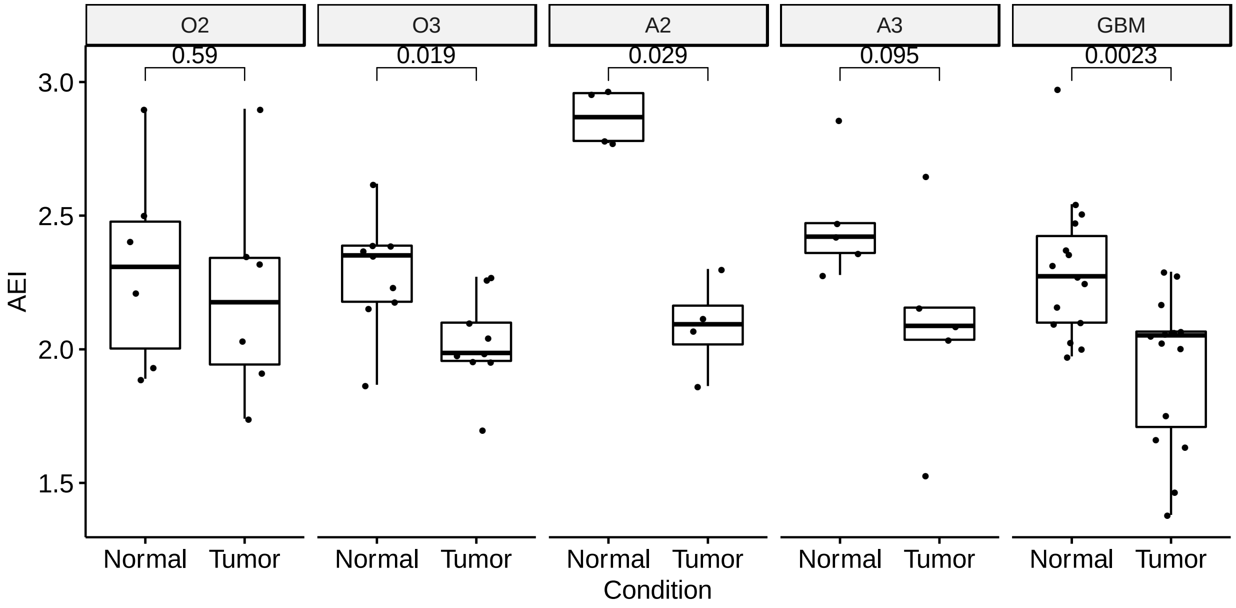

Supplementary Figure 6. Fold change of an RNA polymerase III subunit (*POLR3A*) expression level. Fold changes (Tumor/Normal) of RNA expression (measured by RNA-seq as transcripts per million in log2 scale) were plotted. A dot is a single patient. *P-values* of comparing tumor and matched normal tissues by a linear regression model controlling patient-specific effects were indicated at top (N=12, 18, 8, 10, 4, 30 tissues for O2, O3, A2, A3, A4, GBM, respectively). In a boxplot, the whiskers extend from the bottom and top of the box (the first and third quartiles) to the largest and the smallest value no further than 1.5 * inter-quartile range.

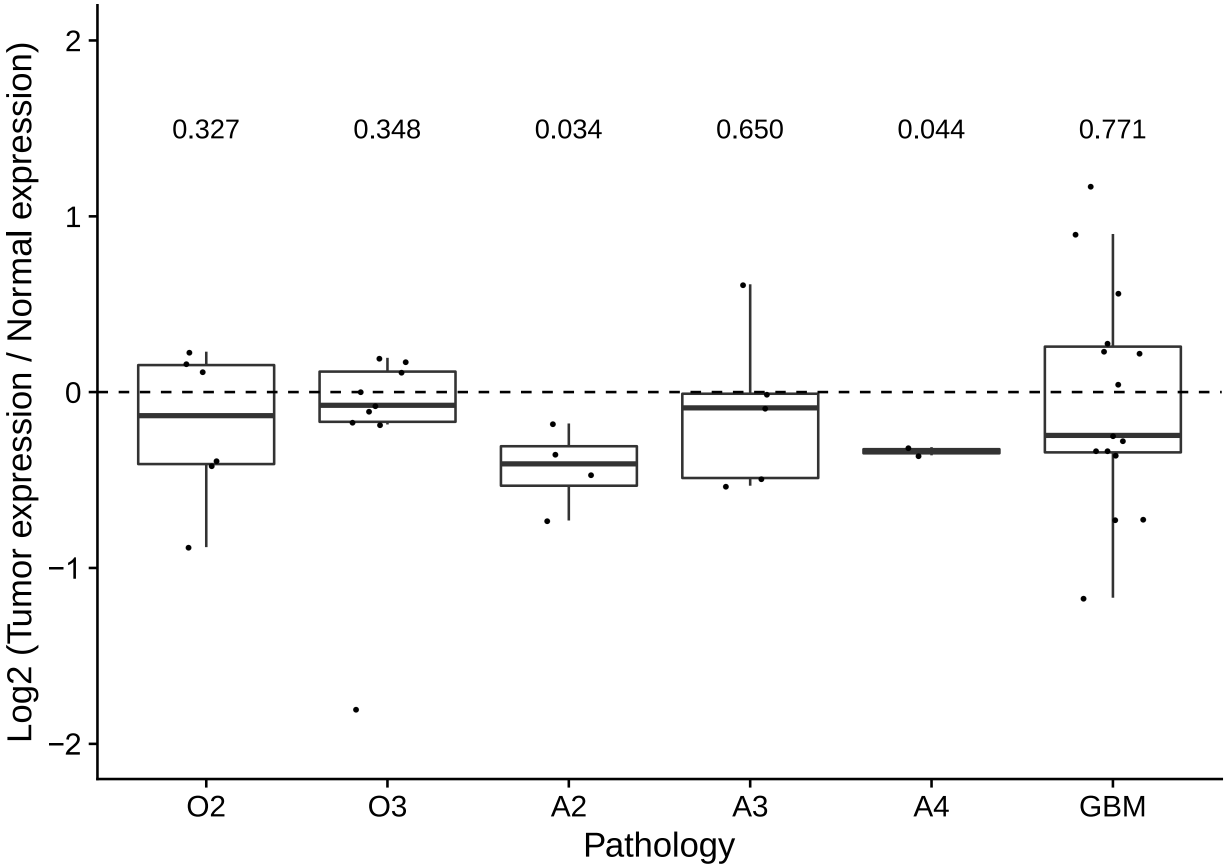

Supplementary Figure 7. Gene ontology analysis of genes with perturbed circular RNA rates. Gene ontology terms that were enriched with the genes whose gene bodies harbor the perturbed circular RNA rate between tumor and matched normal tissues. The terms found in at least two pathologies of glioma were shown.

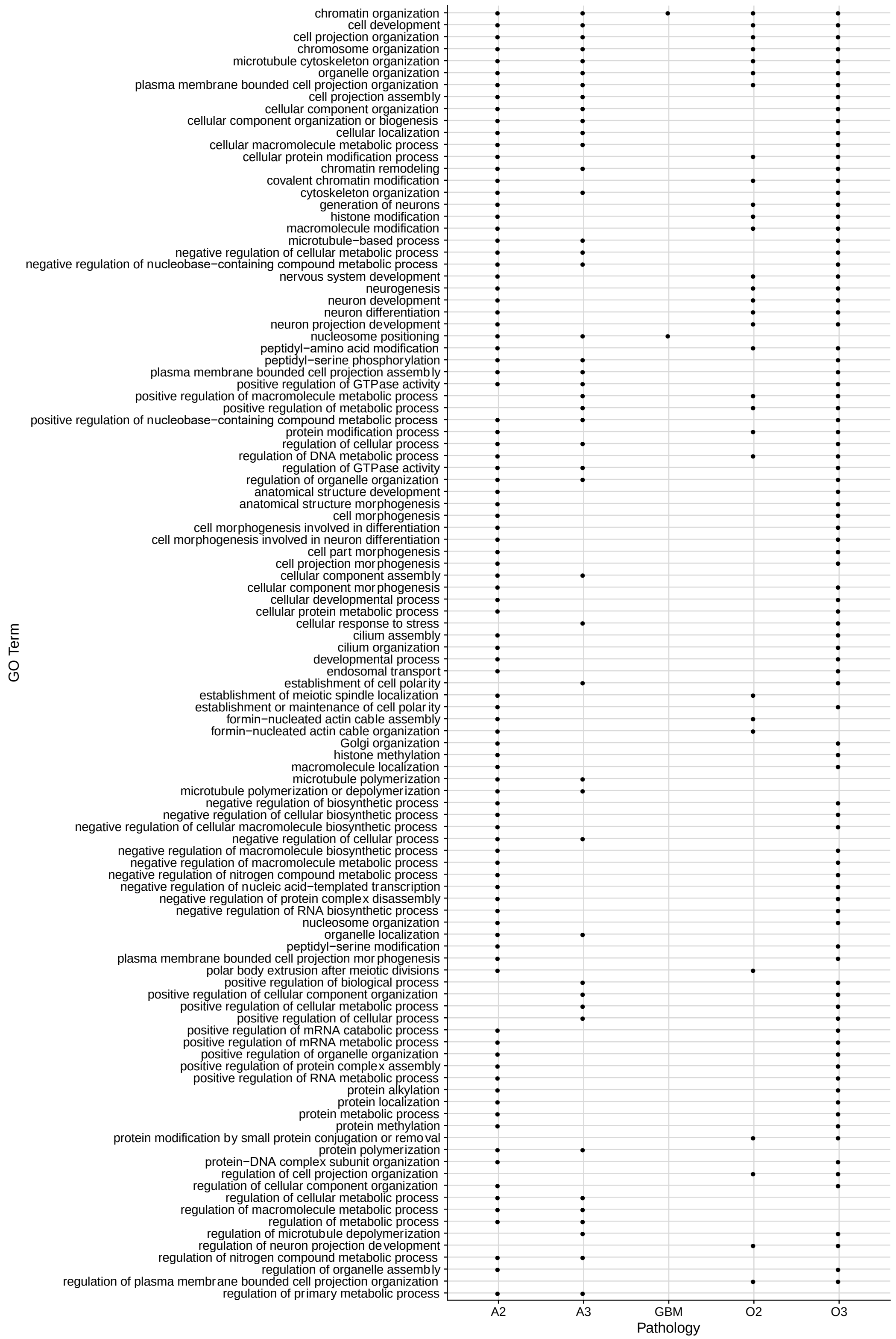

Supplementary Table 1. Table of the samples in the current dataset. *Sex* is either M (male) or F (female), *Age* unit is years, *Grade* is WHO grade, *Tissue* is an indicator of either normal tissue or tumor tissue of a sample, *Pathology* is an acronym of diagnosis: grade 2 oligodendroglioma *IDH* mutant and 1p/19q-codeleted (O2), grade 3 oligodendroglioma *IDH* mutant and 1p/19q-codeleted (O3), grade 2 *IDH* mutant astrocytoma (A2), grade 3 *IDH* mutant astrocytoma (A3), grade 4 *IDH* mutant astrocytoma (A4), and glioblastoma (GBM). *TotalNum* is the total number of sequencing reads.

Patient ID	Sex	Age	Grade	Pathology	Tissue	Sample ID	TotalNum
Case_1	M	31	2	O2	Normal	N35	50,313,037
Case_1	M	31	2	O2	Tumor	T35	94,090,964
Case_2	F	40	2	O2	Normal	N37	90,531,827
Case_2	F	40	2	O2	Tumor	T37	76,628,565
Case_3	F	41	2	O2	Normal	N38	50,590,460
Case_3	F	41	2	O2	Tumor	T38	64,829,220
Case_44	M	44	2	O2	Normal	N54	62,044,812
Case_44	M	44	2	O2	Tumor	T54	54,938,487
Case_45	M	57	2	O2	Normal	N55-2	60,034,724
Case_45	M	57	2	O2	Tumor	T55	61,563,871
Case_46	M	34	2	O2	Normal	N48-1	62,895,363
Case_46	M	34	2	O2	Tumor	T56	62,420,477
Case_35	F	53	3	O3	Normal	N43-1	60,582,037
Case_35	F	53	3	O3	Tumor	T43-1	63,068,587
Case_36	M	65	3	O3	Normal	N44	59,085,907
Case_36	M	65	3	O3	Tumor	T44	53,676,699
Case_37	F	38	3	O3	Normal	N45-2	62,174,446
Case_37	F	38	3	O3	Tumor	T45	62,150,861
Case_38	M	58	3	O3	Normal	N46-2	62,130,572
Case_38	M	58	3	O3	Tumor	T46	62,763,517
Case_39	F	67	3	O3	Normal	N47-2	65,538,920
Case_39	F	67	3	O3	Tumor	T47	62,946,268
Case_4	F	38	3	O3	Normal	N31	98,035,122
Case_4	F	38	3	O3	Tumor	T31	81,794,210
Case_41	F	57	3	O3	Normal	N49	62,444,495
Case_41	F	57	3	O3	Tumor	T49	60,964,164
Case_5	M	57	3	O3	Normal	N32	94,141,757
Case_5	M	57	3	O3	Tumor	T32	86,403,961
Case_6	F	34	3	O3	Normal	N33	52,257,079
Case_6	F	34	3	O3	Tumor	T33	68,905,794
Case_42	M	27	2	A2	Normal	N51-1	63,867,364
Case_42	M	27	2	A2	Tumor	T51	72,218,243
Case_43	F	28	2	A2	Normal	N52	64,875,912

Case_43	F	28	2	A2	Tumor	T52	62,947,903
Case_7	F	51	2	A2	Normal	N1	50,969,978
Case_7	F	51	2	A2	Tumor	T1	78,539,368
Case_8	F	47	2	A2	Normal	N2	62,777,288
Case_8	F	47	2	A2	Tumor	T2	54,624,154
Case_11	M	22	3	A3	Normal	N7	59,067,885
Case_11	M	22	3	A3	Tumor	T7	56,972,487
Case_13	M	35	3	A3	Normal	N8	51,155,356
Case_13	M	35	3	A3	Tumor	T8	57,100,211
Case_19	F	49	4	A3	Normal	N17	60,538,741
Case_19	F	49	4	A3	Tumor	T17	73,814,874
Case_31	F	34	3	A3	Normal	N39	61,264,620
Case_31	F	34	3	A3	Tumor	T39	62,880,242
Case_33	F	43	3	A3	Normal	N41	60,107,576
Case_33	F	43	3	A3	Tumor	T41	56,105,186
Case_26	F	30	4	A4	Normal	N26	54,138,088
Case_26	F	30	4	A4	Tumor	T26	61,944,670
Case_27	F	42	4	A4	Normal	N27	57,152,911
Case_27	F	42	4	A4	Tumor	T27	61,199,405
Case_10	M	54	3	GBM	Normal	N6	64,771,502
Case_10	M	54	3	GBM	Tumor	T6	67,438,698
Case_17	M	52	4	GBM	Normal	N12	62,066,884
Case_17	M	52	4	GBM	Tumor	T12	50,837,787
Case_18	F	65	4	GBM	Normal	N13	62,002,377
Case_18	F	65	4	GBM	Tumor	T13	56,367,364
Case_20	M	73	4	GBM	Normal	N18	88,276,825
Case_20	M	73	4	GBM	Tumor	T18	58,559,093
Case_21	M	55	4	GBM	Normal	N19	73,101,680
Case_21	M	55	4	GBM	Tumor	T19	51,738,071
Case_22	M	49	4	GBM	Normal	N20	64,839,051
Case_22	M	49	4	GBM	Tumor	T20	56,363,802
Case_23	M	59	4	GBM	Normal	N22	53,564,250
Case_23	M	59	4	GBM	Tumor	T22	84,523,167
Case_24	M	40	4	GBM	Normal	N24	65,677,054
Case_24	M	40	4	GBM	Tumor	T24	69,636,477
Case_25	F	71	4	GBM	Normal	N25	61,976,660
Case_25	F	71	4	GBM	Tumor	T25	71,737,747
Case_28	M	68	4	GBM	Normal	N14	52,334,098

Case_28	M	68	4	GBM	Tumor	T14	74,045,443
Case_29	F	52	4	GBM	Normal	N16	84,490,686
Case_29	F	52	4	GBM	Tumor	T16	56,338,203
Case_30	M	38	4	GBM	Normal	N21	51,827,007
Case_30	M	38	4	GBM	Tumor	T21	58,218,272
Case_32	F	52	3	GBM	Normal	N40	58,642,093
Case_32	F	52	3	GBM	Tumor	T40	51,066,242
Case_34	M	56	3	GBM	Normal	N42-1	64,671,576
Case_34	M	56	3	GBM	Tumor	T42	60,589,929
Case_9	M	55	3	GBM	Normal	N4	66,208,530
Case_9	M	55	3	GBM	Tumor	T4	56,983,635
