# Supplementary material for: Genome-wide perturbations of Alu expression and Alu-associated post-transcriptional regulations distinguish oligodendroglioma from other gliomas
Source: Commun Biol. 2022 Jan 18;5:62. doi: 10.1038/s42003-022-03011-w (PMC8766575; doi:10.1038/s42003-022-03011-w)
Supplement: Supplementary file 3 — Description of Additional Supplementary Files [file 42003_2022_3011_MOESM3_ESM.pdf]

Description of Additional Supplementary Files

File Name: Supplementary Data 1.
Description: The A-to-I editing sites shared by all the patients. Pos: hg38 coordinate. Strand: strand determined by RNA-seq. Alu: 1/associated, 0/non-associated. All the other columns (Patient ID with suffix of _N: normal, _T: tumor): A-to-I editing level per sample.

File Name: Supplementary Data 2.
Description: The perturbed A-to-I editing sites between tumor and normal tissues. Pathology: grade 2 oligodendroglioma *IDH* mutant and 1p/19q-codeleted (O2), grade 3 oligodendroglioma *IDH* mutant and 1p/19q-codeleted (O3), grade 2 *IDH* mutant astrocytoma (A2), grade 3 *IDH* mutant astrocytoma (A3), and glioblastoma (GBM). Pos: hg38 coordinate. Exonic: 1/associated, 0/non-associated. Alu: 1/associated, 0/non-associated. Diff: A-to-I editing level difference between tumor and normal (tumor-normal). Padj: FDR-adjusted p-value.

File Name: Supplementary Data 3.
Description: The list of genes with perturbed circular RNA expression rates between tumor and normal tissues. Gene_id: Gencode gene id**.** Gene_name: gencode gene name. Pathology: grade 2 oligodendroglioma *IDH* mutant and 1p/19q-codeleted (O2), grade 3 oligodendroglioma *IDH* mutant and 1p/19q-codeleted (O3), grade 2 *IDH* mutant astrocytoma (A2), grade 3 *IDH* mutant astrocytoma (A3), and glioblastoma (GBM). Pos: hg38 coordinate. Exonic: 1/associated, 0/non-associated. CircMedian: the median of circular RNA-supporting (backsplicing) junction read counts. JReadMedian: the median of canonical splicing junction read counts. RateTumor and RateNormal: circular RNA expression rate in tumor and normal tissue, respectively. Padj: FDR-adjusted p-value.

File Name: Supplementary Data 4.
Description: The result of gene ontology analysis for the genes with the perturbed A-to-I editing in gliomas. Pathology: grade 3 oligodendroglioma *IDH* mutant and 1p/19q-codeleted (O3), grade 2 *IDH* mutant astrocytoma (A2), grade 3 *IDH* mutant astrocytoma (A3), and glioblastoma (GBM). GOBPID: Gene ontology biological process term ID. Term: Gene ontology term, Pvalue: p-value calculated by R package GOstats.

File Name: Supplementary Data 5.
Description: The result of gene ontology analysis for the genes with the perturbed circular RNA expression rate. Pathology: grade 2 oligodendroglioma *IDH* mutant and 1p/19q-codeleted (O2), grade 3 oligodendroglioma *IDH* mutant and 1p/19q-codeleted (O3), grade 2 *IDH* mutant astrocytoma (A2), grade 3 *IDH* mutant astrocytoma (A3), and glioblastoma (GBM). GOBPID: Gene ontology biological process term ID. Term: Gene ontology term, Pvalue: p-value calculated by R package GOstats.

File Name: Supplementary Data 6.
Description: The proportion of RNA variants detected in the samples. This is the raw data of the main figure 1a. Number: the number of the corresponding variant sites.

File Name: Supplementary Data 7.
Description: The summary of A-to-I editing and circular RNA. This is the raw data for the main figures 1b, 1d and 2b. MappedReadNum: the total number of RNA-seq reads mapped to the reference genome by our computational pipeline. AtoIeditingNum: the number of A-to-I editing sites. AEI: Alu Editing Index. CircReadNum: the number of RNA-seq junction reads with circular RNA feature (back-splicing).

File Name: Supplementary Data 8.
Description: The association of gene with *Alu* element and circular RNA. This is the raw data for the main figures 2a. Gene_id and Gene name: GENCODE (v27) information. Alu: whether a gene is overlapped with *Alu* element (0: no overlap, 1: overlap). CircFound: whether circular RNAs are detected for a gene (FALSE: undetected, TRUE: detected).

File Name: Supplementary Data 9.
Description: Differential expression analysis of 47 Alu subfamilies per pathologies. This is the raw data for the main figures 3a and 3c. Subfamily name: the name of alu subfamily. Pathology: grade 2 oligodendroglioma *IDH* mutant and 1p/19q-codeleted (O2), grade 3 oligodendroglioma *IDH* mutant and 1p/19q-codeleted (O3), grade 2 *IDH* mutant astrocytoma (A2), grade 3 *IDH* mutant astrocytoma (A3), and glioblastoma (GBM). BaseMean, Log2FoldChange, Pvalue and Padj: the output of DESeq2 (DESeq2-normalized mean read counts, log2 fold change of tumor relative to normal tissues, p-value and FDR-adjusted p-value).

File Name: Supplementary Data 10.
Description: Differential expression analysis of *ADAR* families per pathologies. This is the raw data for the main figures 4a. Gene name: the name of gene. Pathology: grade 2 oligodendroglioma *IDH* mutant and 1p/19q-codeleted (O2), grade 3 oligodendroglioma *IDH* mutant and 1p/19q-codeleted (O3), grade 2 *IDH* mutant astrocytoma (A2), grade 3 *IDH* mutant astrocytoma (A3), and glioblastoma (GBM). BaseMean, Log2FoldChange, Pvalue and Padj: the output of DESeq2 (DESeq2-normalized mean read counts, log2 fold change of tumor relative to normal tissues, p-value and FDR-adjusted p-value).
