# Supplementary material for: Genome-wide perturbations of Alu expression and Alu-associated post-transcriptional regulations distinguish oligodendroglioma from other gliomas
Source: Commun Biol. 2022 Jan 18;5:62. doi: 10.1038/s42003-022-03011-w (PMC8766575; doi:10.1038/s42003-022-03011-w)
Supplement: Supplementary file 14 — Reporting Summary [file 42003_2022_3011_MOESM14_ESM.pdf]

nature research

Corresponding author(s): Park, Chul-Kee and Hwang, Taeyoung

Last updated by author(s): Dec 6, 2021

Reporting Summary

Nature Research wishes to improve the reproducibility of the work that we publish. This form provides structure for consistency and transparency in reporting. For further information on Nature Research policies, see our Editorial Policies and the Editorial Policy Checklist.

Statistics

For all statistical analyses, confirm that the following items are present in the figure legend, table legend, main text, or Methods section.

n/a	Confirmed	
☐	☒	The exact sample size (n) for each experimental group/condition, given as a discrete number and unit of measurement
☐	☒	A statement on whether measurements were taken from distinct samples or whether the same sample was measured repeatedly
☐	☒	The statistical test(s) used AND whether they are one- or two-sided<br>*Only common tests should be described solely by name; describe more complex techniques in the Methods section.*
☐	☒	A description of all covariates tested
☐	☒	A description of any assumptions or corrections, such as tests of normality and adjustment for multiple comparisons
☐	☒	A full description of the statistical parameters including central tendency (e.g. means) or other basic estimates (e.g. regression coefficient) AND variation (e.g. standard deviation) or associated estimates of uncertainty (e.g. confidence intervals)
☐	☒	For null hypothesis testing, the test statistic (e.g. F, t, r) with confidence intervals, effect sizes, degrees of freedom and P value noted<br>*Give P values as exact values whenever suitable.*
☒	☐	For Bayesian analysis, information on the choice of priors and Markov chain Monte Carlo settings
☒	☐	For hierarchical and complex designs, identification of the appropriate level for tests and full reporting of outcomes
☐	☒	Estimates of effect sizes (e.g. Cohen's d, Pearson's r), indicating how they were calculated

Our web collection on statistics for biologists contains articles on many of the points above.

Software and code

Policy information about availability of computer code

Data collection	NA
Data analysis	STAR (version: 2.6.0c), Picard MarkDuplicates (version: 2.6.0), FeatureCounts (version: subread 1.6.2), R packages Rtsne (version: 0.15) and GOstats, R functions from Redit (https://github.com/gxiaolab/REDITs), CIRCexplorer2 (version: 2.3.8), TEtranscripts (version: 2.1.4).

For manuscripts utilizing custom algorithms or software that are central to the research but not yet described in published literature, software must be made available to editors and reviewers. We strongly encourage code deposition in a community repository (e.g. GitHub). See the Nature Research guidelines for submitting code & software for further information.

Data

Policy information about availability of data

All manuscripts must include a data availability statement. This statement should provide the following information, where applicable:

- Accession codes, unique identifiers, or web links for publicly available datasets
- A list of figures that have associated raw data
- A description of any restrictions on data availability

Raw RNA-seq data can be accessed through NCBI GEO (GSE165595).

Field-specific reporting

Please select the one below that is the best fit for your research. If you are not sure, read the appropriate sections before making your selection.

☒ Life sciences ☐ Behavioural & social sciences ☐ Ecological, evolutionary & environmental sciences

For a reference copy of the document with all sections, see nature.com/documents/nr-reporting-summary-flat.pdf

Life sciences study design

All studies must disclose on these points even when the disclosure is negative.

Sample size	The sample size was restricted by available tissues from glioma patients who underwent surgery at Seoul National University Hospital, Seoul, South Korea.
Data exclusions	After performing stranded RNA-seq, we removed one patient for further analysis as its tumor sample has lower sequencing quality based on the number of counted reads for gene expression measurement (lower than 10% of total sequencing reads).
Replication	We focused on the A-to-I editing sites that found in multiple patients. Also, statistical comparisons were done with the measurements from multiple patients.
Randomization	N/A. Patient tissues were obtained durring surgical treatment. All tissues were uniformly processed.
Blinding	N/A. A matched pair of tumor and normal samples were obtained from every 42 patients.

Reporting for specific materials, systems and methods

We require information from authors about some types of materials, experimental systems and methods used in many studies. Here, indicate whether each material, system or method listed is relevant to your study. If you are not sure if a list item applies to your research, read the appropriate section before selecting a response.

Materials & experimental systems

n/a	Involved in the study	
☒	☐	Antibodies
☒	☐	Eukaryotic cell lines
☒	☐	Palaeontology and archaeology
☒	☐	Animals and other organisms
☐	☒	Human research participants
☒	☐	Clinical data
☒	☐	Dual use research of concern

Methods

n/a	Involved in the study	
☒	☐	ChIP-seq
☒	☐	Flow cytometry
☒	☐	MRI-based neuroimaging

Human research participants

Policy information about studies involving human research participants

Population characteristics	Pathology results of patients are provided in the supplementary table.
Recruitment	Surgical specimens and clinical information were obtained from glioma patients who underwent surgery at Seoul National University Hospital, Seoul, South Korea.
Ethics oversight	This study was performed under the approval of the Institutional Review Board of Seoul National University Hospital, Seoul, South Korea (IRB approval No., H-1404-056-572).

Note that full information on the approval of the study protocol must also be provided in the manuscript.
